# β-Amyloid triggers aberrant over-scaling of homeostatic synaptic plasticity

**DOI:** 10.1186/s40478-016-0398-0

**Published:** 2016-12-13

**Authors:** James Gilbert, Shu Shu, Xin Yang, Youming Lu, Ling-Qiang Zhu, Heng-Ye Man

**Affiliations:** 1 Department of Biology, Boston University, Boston, MA USA; 2 Department of Physiology, School of Basic Medicine, Tongji Medical College, Huazhong University of Science and Technology, Wuhan, 430030 People’s Republic of China; 3 Department of Pathophysiology, School of Basic Medicine, Tongji Medical College, Huazhong University of Science and Technology, Wuhan, 430030 People’s Republic of China; 4 Department of Pharmacology & Experimental Therapeutics, Boston University School of Medicine, Boston, MA USA; 5 The Institute for Brain Research, Collaborative Innovation Center for Brain Science, Huazhong University of Science and Technology, Wuhan, 430030 People’s Republic of China

**Keywords:** Homeostatic synaptic plasticity, Amyloid beta, Microrna 124, Calcium permeable AMPA receptor

## Abstract

The over-production of β-amyloid (Aβ) has been strongly correlated to neuronal dysfunction and altered synaptic plasticity in Alzheimer’s disease (AD). Accordingly, it has been proposed that disrupted synaptic transmission and neuronal network instability underlie memory failure that is evident in the early phases of AD. Homeostatic synaptic plasticity (HSP) serves to restrain neuronal activity within a physiological range. Therefore a disruption of this mechanism may lead to destabilization in synaptic and neural circuit function. Here, we report that during HSP by neuronal activity deprivation, application of Aβ results in an aberrant over-response of the up-regulation of AMPA receptor (AMPAR)-mediated synaptic currents and cell-surface AMPAR expression. In the visual cortex, in vivo HSP induced by visual deprivation shows a similar over-response following an Aβ local injection. Aβ increases the expression of GluA2-lacking, calcium permeable AMPARs (CP-AMPARs), which are required for the initiation, but not maintenance of HSP. Both GluA2-lacking and GluA2-containing AMPARs contribute to the Aβ-mediated over-scaling of HSP. We also find that Aβ induces the dissociation of HDAC1 from the miR124 transcription factor EVI1, leading to an up-regulation of miR124 expression and increased amount of CP-AMPARs. Thus, *via* aberrant stimulation of miR124 expression and biogenesis of CP-AMPARs, Aβ is able to induce an over response in HSP. This Aβ-mediated dysregulation in homeostatic plasticity may play an important role in the pathogenesis of altered neural function and memory deficits in the early stages of AD.

**Electronic supplementary material:**

The online version of this article (doi:10.1186/s40478-016-0398-0) contains supplementary material, which is available to authorized users.

## Introduction

Alzheimer’s Disease (AD) is characterized by deficits in learning and memory with an eventual loss of higher cognitive functions. Accumulation of β-amyloid (Aβ) in the brain is a hallmark of AD and studies have demonstrated that Aβ can induce synapse dysregulation and altered neuronal activity [26, 32, 37, 54, 58]. Emerging evidence suggests that soluble Aβ oligomers adversely affect synaptic function, which eventually leads to the cognitive failure associated with AD [36, 43, 48, 58]. A key neuropathobiological hallmark in early AD is the aberrant regulation in synaptic function including AMPA receptor (AMPAR) synaptic accumulation and synaptic plasticity [7, 43]. In vitro studies performed in hippocampal neurons have reported that application of Aβ peptides, at concentrations below neurotoxic levels, can inhibit LTP induction without affecting basal synaptic transmission [8, 9, 61]. A similar result was shown in vivo, where cerebral injection of naturally secreted Aβ collected from cells expressing amyloid precursor protein (APP) prevented the stable maintenance of LTP in the hippocampal CA1 region [58]. In vivo injection of Aβ is reported to facilitate LTD and LTP reversal (depotentiation) in the CA1 region of the hippocampus [32]. These studies have provided great insight into Hebbian plasticity in AD, however the role of Aβ in homeostatic synaptic plasticity (HSP) remains largely unknown [29].

A major function of HSP is to regulate neuronal activity in a negative feedback manner, thus maintaining neuronal activity or synaptic function [15, 23] within a physiological range after changes in network activity [6, 10, 55, 56]. Under chronic suppression of neuronal activity, HSP is expressed *via* an increase in synaptic expression of AMPARs producing an up-scaling of AMPAR-mediated miniature post-synaptic currents (mEPSCs). While most studies show inactivity-induced synaptic scaling in cultured neurons [2, 46, 50, 55], HSP is also observed in vivo including in the spinal cord [16, 19, 33, 59] and in the visual cortex [11, 17, 31, 34, 38]. AMPARs are heterotetrameric ion channels consisting of different compositions of the four subunits GluA1–4, and the most common of which are GluA1/GluA2 and GluA2/GluA3 combinations [5, 13]. During the early phase of neural inhibition, a change in GluA2 expression leads to the formation of GluA2-lacking, calcium permeable AMPARs (CP-AMPARs). The production and insertion of CP-AMPARs at the synapse is required for the initiation of HSP. We have recently shown that the brain-enriched microRNA, miR124, causes a selective reduction in GluA2 levels *via* interaction with its 3’-UTR, leading to CP-AMPAR expression and HSP [24].

Here we report that during inactivity-dependent HSP, either in vitro in cultured neurons with TTX incubation or in vivo in the visual cortex with visual deprivation, application of Aβ results in an aberrant over-scaling of AMPAR-mediated synaptic currents and surface AMPAR expression. Aβ incubation or brain injection produces the expression of additional CP-AMPARs under neuronal activity inhibition. The CP-AMPARs are required for the initiation, but not maintenance of HSP. Consistent with this, both in vitro in cultured neurons with TTX incubation, and in vivo in the visual cortex during visual deprivation, application of Aβ leads to increased miR124 expression and the Aβ-mediated HSP can be blocked by miR124 suppression. Additionally, we show that Aβ induces the dissociation of HDAC1 from the inhibitory miR124 transcription factor EVI1, producing an up-regulation of miR124 expression and increased generation of CP-AMPARs. Thus, Aβ induces an over-response to inactivity-dependent HSP *via* an upregulation of miR124 and CP-AMPAR expression. Therefore in the presence of Aβ, neurons adapt their synaptic properties distinctly, which is likely to cause destabilization in neural network operation and brain function in AD.

## Materials and methods

### Drugs, antibodies and plasmids

TTX, APV and PhTx were purchased from Sigma Aldrich and prepared in water. Bicuculline was purchased from Tocris Bioscience and prepared in DMSO. All drugs were prepared in a 1000× stock solution, stored in aliquots at −20°C and thawed only once prior to use to preserve their potency. GluA1N and GluA2N antibodies (1:500, Mouse, Millipore) GluA1C (1:500, Rabbit, homemade) were used for staining AMPARs and probed with Alexa-fluor conjugated secondary antibodies (1:700, Invitrogen). EVI1 (Rabbit, Abcam) and HDAC1 (Mouse, Cell Signaling) were used for co-immunoprecipitation (1μg) and western blotting (1:1000) experiments. Aβ (Rabbit, U6590) was a generous gift from the Angela Ho Lab, Boston University. Two tandem repeats of the miR124-BS sequence (5’-TGGCATTCACAAGTGCCTTAA -3’) were cloned into pEGFP-N1. Lentivirus-encoding a reverse complementary sequence of miR-124 was purchased from Genechem Inc (Shanghai, China).

β*-Amyloid.* Synthetic Aβ [1–42] was purchased from Invitrogen and prepared according to the manufacturer’s instructions. Briefly, the peptide was dissolved in HPLC grade water at 1mM then diluted to 200 μM in PBS and incubated at 37°C for 24 h. Samples were aliquoted, stored at −20°C, and thawed once directly prior to use.

### Neuronal cultures

Primary hippocampal cultures were prepared from embryonic day 18 rat embryos as previously described [40]. Cells (0.4–0.6 × 10^6^) were plated into a 60-mm dish with polylysine-precoated coverslips and maintained in neurobasal medium that was replenished twice a week. Neurons were treated and recorded between 14–16 days in vitro (DIV).

### Aβ and drug treatment

Aβ (0.5 μM), TTX (1 μM) or APV (25 μM) were added directly to the culture medium at 14–15 DIV and allowed to incubate at 37°C for the time specified prior to recording. For recovery experiments, neurons were treated for 24 h with Aβ and/or TTX, washed 3× with artificial cerebrospinal fluid (ACSF) and fresh culture medium was replaced for the time indicated.

### Immunocytochemistry

Neurons were washed with ACSF and fixed with 4% paraformaldehyde/4% sucrose for 10 minutes, and stained without permeabilization for surface labeling. Coverslips with neurons were blocked with 10% goat serum in ACSF for 1 h and then incubated with the GluA1-N terminal and GluA2-N terminal antibodies dissolved in 5% goat serum in ACSF for 2 h at room temperature. Cells were washed three times with PBS and incubated with fluorescent Alexa-fluor-conjugated secondary antibodies for 1 h prior to visualization. Coverslips were mounted onto slides with Prolong Gold Anti-fade Reagent for visualization on the microscope.

### Cell death assay

After undergoing the different HSP paradigms, neurons were treated with propidium iodide (PI, 1μg/mL, Sigma-Aldrich) to label the nuclei of dying cells and Hoescht (1μg/mL, Cell Signaling) to label all nuclei. Both PI and Hoescht were added at the same time, directly to the culture media, and allowed to incubate for 20 min at 37°C. As a positive control, one set of neurons was treated with glutamate (30 μM) for 30 min, washed with ACSF and the media replaced and returned to the incubator for 3 hours prior to PI and Hoescht labeling. Cells were then washed with ACSF and fixed with 4% paraformaldehyde/4% sucrose for 10 minutes. Following a wash with PBS, neuronal coverslips were mounted onto slides with Prolong Gold Anti-fade Reagent for microscopy. The ratio of PI-positive nuclei to total nuclei (Hoescht) was calculated to determine the percentage of dying cells.

### Image collection and analysis

Mounted coverslips were kept overnight in the dark at room temperature before imaging with a 63× oil-immersion objective. A DIC image was taken for morphology purposes and images were collected with Axiovision 4.8 software and exposure time for the fluorescence signal was adjusted manually so the signals were within a full dynamic range. Either the glow scale look-up table or the histogram was used to monitor the saturation level. Once the parameters were set, they were fixed and used throughout the experiment. Typically, —three to five sections of proximal dendrites per cell were used for analysis. AMPAR clusters were individually inspected to avoid contamination with nonspecific signals.

For accurate quantification, all images were collected in 8-bit gray scale and saved for raw data analysis with Image J software.

A multi-colored image (red and green from stained glutamate receptors and a DIC image for morphology) was separated into three channels with Image-J software, and the windows were synchronized. By tracing a neuron’s dendrites in the DIC channel, the corresponding postsynaptic AMPAR clusters were able to be precisely located in the red and green channels..

AMPAR puncta size was calculated as the product of size and fluorescence intensity of each puncta. The data were presented as averages of AMPAR puncta ± SEM normalized to controls. Greater than 100 hundred puncta were measured per cell.

### Co-immunoprecipitation and western blot

Two-week-old cultured cortical neurons were incubated with Aβ and/or TTX for 24 h and harvested in ice-cold RIPA lysis buffer (40 mM Tris–HCl, 150 mM Nacl, 1% NP-40, 1% sodium deoxycholate, 0.1% SDS and a protease inhibitor cocktail containing AEBSF, Aprotinin, Bedysyin, E-64, Leupeptin and Pepstatin A, Roche) and rotated at 4 °C for 45 min. Following centrifugation of the lysates at 13,000g for 15 min, supernatants were incubated overnight on rotation at 4 °C with anti-EVI1 antibodies, (1 μg, Abcam) followed by the addition of 60 μl of 50% slurry of protein A-Sepharose beads (Santa Cruz Biotechnology). Immunoprecipitates were washed three times with lysis buffer and resuspended in 60 μl of 2 × Laemmli buffer and denatured on a 95 °C heat block for 10 min. Immunoprecipitates were analysed by western blotting. For western blot of Aβ species, prepared Aβ oligomers were run on 4–20% gradient TGX gels (Biorad) and transferred to PVDF membranes.

### Electrophysiology

For in vitro mEPSC recordings, 14–15 DIV cultured hippocampal neurons were treated with TTX (1μM) for 24 h to induce HSP. The coverslip was then transferred to a recording chamber with an extracellular solution containing (in mM): 140 NaCl, 3 KCl, 1.5 MgCl_2_, 2.5 CaCl_2_, 11 glucose, and 10 HEPES (305 mOsm, pH 7.4,), which was supplemented with TTX (1μM) to block action potentials, APV (50 μM) to block NMDARs and bicuculline (20 μM) to block GABA_A_ receptor-mediated currents. To block CP-AMPARs, recordings were made in the presence of Philanthotoxin (PhTx, 2 μM) in the extracellular solution. Whole cell voltage-clamp recordings were made with patch pipettes filled with intracellular solution containing (in mM): 110 Cs-methanesulfonate, 10 CsCl, 10 Hepes, 0.2 EGTA, 4 Mg-ATP, 0.3 Na_2_-GTP, and 10 sodium phosphocreatine (295 mOsm, pH 7.4), with the membrane potential clamped at −70mV. Recordings started 5 minutes after establishing whole-cell configuration to ensure equilibration between the pipette solution and the cytosol. Series resistance for recordings ranged between 10–15 MΩ and cell capacitance values were between 85–150 pF. mEPSCs were recorded with an Axopatch 200B amplifier and displayed and recorded digitally on a computer for subsequent off-line analysis with Clampfit.

Templates for a typical mEPSC were created using Clampfit software that averaged 25 manually selected typical events. The templates were used to scan the traces for events with highly similar kinetics and a moving baseline was created from each event to calculate amplitude. Data was then manually inspected for selection of typical mEPSC events. All data were presented as normalized mean ± SEM. For cumulative probabilities, the relationship between control and experimental amplitude and inter-event interval distributions were determined by ranking an equal number of randomly selected values from all cells recorded from each condition in ascending order. These amplitude distributions were linearly interpolated to plot each condition.

To calculate the ratio of CP-AMPAR current to total AMPAR current, the amplitude of neurons recorded without PhTx was subtracted from the amplitude of recordings in the presence of PhTx and divided by the amplitude of recordings performed without PhTx. To calculate N-AMPAR current, the amplitudes of recordings in the presence of PhTX was used.

For in vivo recordings, brain slice sections (300 μm) of the visual cortex (V1 area) were prepared using a vibratory microtome. Slices were allowed to recover at 32°C for 30 min then at room temperature for at least 1 h. The composition of ACSF (in mM) was 124 NaCl, 3 KCl, 1.25 NaH2PO4, 1.2 MgCl2, 2 CaCl2, 26 NaHCO3, and 10 dextrose (305 mOsm, pH 7.4). Brain slices were transferred to a recording chamber and continuously superfused with ACSF (2 ml/min) saturated with 95% O_2_ and 5% CO_2_ supplemented with TTX (1μM), APV (50μM) and bicuculline (20μM) and maintained at room temperature. To block calcium-permeable AMPARs, philanthotoxin (5 μM) was added into the extracellular solution.

Brain slices were visualized with IR-DIC using an Axioscope 2FS equipped with Hamamatsu C2400-07E optics. Recording electrodes were filled with an internal solution containing (in mM): 140 K-gluconate, 10 Hepes, 0.2 EGTA, 2 NaCl, 2 MgATP, 0.3 NaGTP Miniature events from each trace (>300 consecutive events) were identified using template matching in Clampfit software (Molecular Devices) with a threshold of 5 pA, 20%–80% rise time of less than 1 ms and manually confirmed for analysis. All data were acquired at 10 kHz and filtered with a lowpass filter at 2 kHz.

### Visual deprivation

Mice (30 ± 5 days old) were anesthetized with 6% chloral hydrate (6ml/kg; i.p.) and the eyelids were covered with a black pellicle of appropriate size. Black ink powder is mixed with an epoxy adhesive (AB gel) to bond the black pellicle to the closed eyelids. The adhesive was applied across the neck, avoiding the leg, to stabilize the pellicle. After waking up, a powerful light was used to make sure that the visual deprivation procedure was successful. All mice were held for 24 h in a darkroom prior to electrophysiological recordings. Control animals were raised with a normal light cycle (12 h light/dark). Mice were injected with Aβ oligomers (10 μg) in the V1 region (ML ±2.3 mm, AP 3.16 mm, DV 0.9 mm) followed by binocular visual deprivation [3].

### Total RNA extraction and quantitative real-time PCR

Total RNA was purified using TRIzol (Thermo Fisher) and treated with DNAseI (Thermo Fisher) to remove DNA contamination from hippocampal cultures. Total RNA was recovered from 60 mm culture dishes and purity was confirmed with an optical density A260/A280 ratio >1.9. To analyze the expression levels of the mature miR124 sequence (5T- UGUGUUCACAGUGGACCUUGAU −3’), 2 μg of total RNA was reverse transcribed and quantitative real-time PCR (qRT-PCR) was performed with a 7300 real-time PCR system (Applied Biosystems) using a TaqMan mir124 assay kit (Applied Biosystems. Cat. #: 4426961). Transcript levels were normalized to the mature U6 snRNA sequence (5’-GTGCTCGCTTCGGCAGCACATATACTAAAATTGGAACGATACAGAGAAGATTAGCATGGCCCCTGCGAGGATGACACGCAAATTCGTGAAGCGTTCCATATT-3’) using a micro RNA Taqman assay (Applied Biosystems, Cat. # 4427975).

### Animals for visual deprivation

Mice (male C57BL/6J mice, 80 ± 2 days older) were raised with a normal light cycle (12 h light/dark). The mice were anesthetized with 6% chloral hydrate (0.06ml/10 g; i.p.). Mice were injected with Aβ oligomers (10 μg) in the V1 region (ML 2.3, AP 3.16, DV 0.9) and then underwent the visual deprived procedure. This study was approved by the Animal Care and Use Committee of Tongji Medical College.

### Statistics

Statistical analysis was performed with GraphPad Prism 6 software using a Kruskal-Wallis non-parametric test to check for significance followed by the Mann–Whitney U to test between group comparisons, with a Bonferoni correction for multiple comparisons where appropriate.

## Results

### Aβ causes over-scaling during inactivity-dependent HSP

**Fig. 1:**
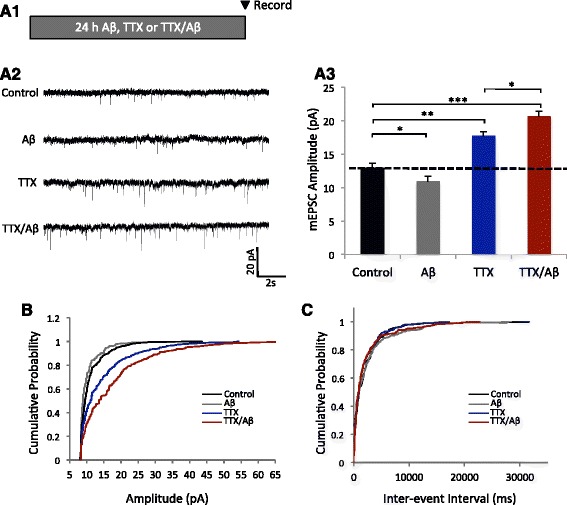
Aβ triggers an aberrant homeostatic response during neuronal activity deprivation. **a1** Treatment paradigm. Neurons were treated with Aβ alone, TTX alone, or TTX and Aβ together (TTX/Aβ) for 24 h prior to mEPSC recordings. **a2** Sample mEPSC traces showing Aβ-mediated HSP. **a3** Quantification of mEPSC amplitudes (control = 12.9 ± 0.75, *n* = 9; Aβ = 10.9 ± 0.79, *n* = 9; TTX = 17.7 ± 0.67, *n* = 7; TTX/Aβ = 20.6 ± 0.82, *n* = 8. **b**, **c** Cumulative probability plots showing no change in inter-event interval (**b**) and a typical multiplicative scaling effect on mEPSC amplitude (**c**). Mann–Whitney *U* test. *p < 0.05, **p < 0.01, ***p < 0.001

To assess the effect of Aβ on HSP during activity deprivation, Aβ oligomers (0.5 μM) were applied to cultured rat hippocampal neurons, with TTX (1 μM) or vehicle (water), for 24 h and AMPAR-mediated mEPSCs were recorded (Fig. 1a1). The Aβ (1–42) monomer was allowed to aggregate into oligomeric species during preparation and confirmed *via* western blot (Additional file 1: Figure S1). In a typical HSP response after TTX incubation, we observed a 39% increase in mEPSC amplitude. Consistent with previous reports, Aβ treatment led to a modest but significant decrease in mEPSC amplitude after 24 h [7, 14]. Strikingly, when Aβ was co-applied together with TTX (TTX/Aβ) in neurons, we found that mEPSCs were increased to approximately 60% in amplitude, a response significantly stronger than a regular HSP response by TTX alone (Fig. 1a2–a3). Additionally, using a different paradigm in which HSP was induced with the application of TTX and the NMDA receptor antagonist APV (25 μM), we found that co-application of Aβ produced a 22% greater increase in mEPSC amplitude compared to treatment with TTX + APV alone (Additional file 1: Figure S2). Therefore, in the presence of Aβ, activity deprivation induced over-scaling of mEPSC during homeostatic regulation. A rightward shift in the cumulative distribution of mEPSC amplitudes (Fig. 1b) was not accompanied by any changes in inter-event interval (Fig. 1c) or frequency (Additional file 1: Figure S3), suggesting a predominant postsynaptic change in AMPA receptor currents.

### Aβ does not alter the set-point of HSP

**Fig. 2:**
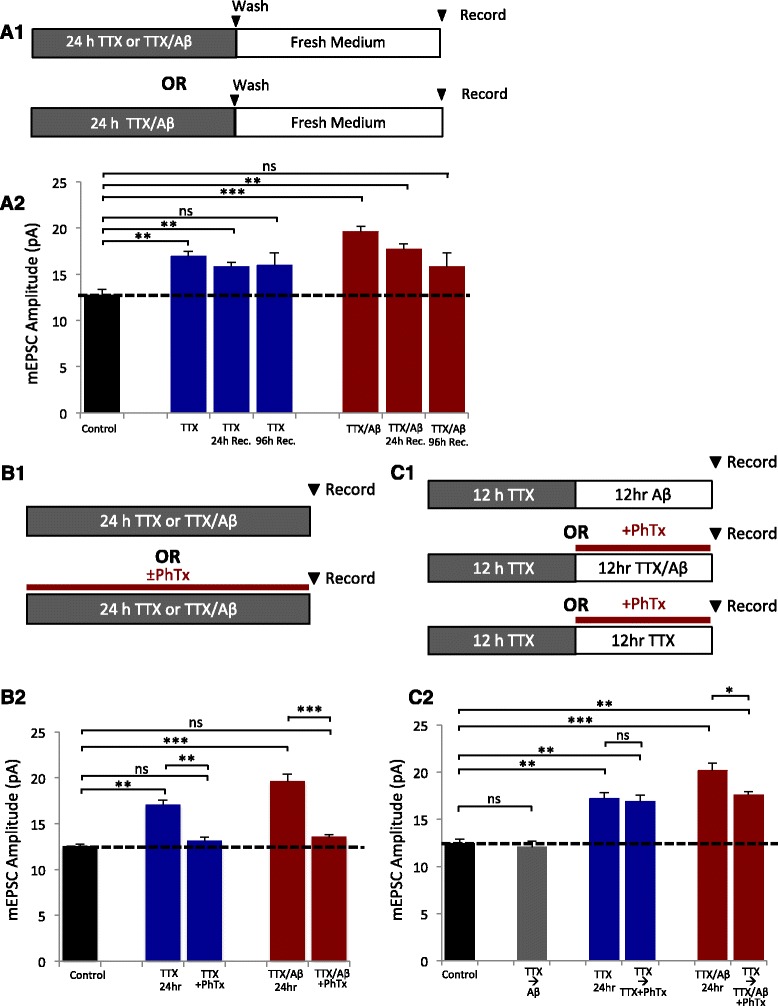
Recovery time course and requirement of CP-AMPARs for HSP. **a1** Treatment paradigm for HSP recovery. Neurons were incubated in the appropriate treatment for 24 h, washed, then allowed to recover in fresh medium for various lengths of time. **a2** Normalized mEPSC amplitudes after recovery for 24 and 96 h. Both the TTX and TTX/Aβ treatment groups returned to baseline after 96 h recovery, indicating a lack of change in the set point of neuronal activity during basal conditions (control = 12.7 ± 0.62, *n* = 9; TTX = 16.9 ± 0.56, *n* = 13; TTX 24 h recovery = 15.8 ± 0.51, *n* = 9; TTX 96 h recovery = 15.9 ± 1.33, *n* = 8; TTX/Aβ = 19.6 ± 0.54, *n* = 10; TTX/Aβ 24 h recovery = 17.7 ± 0.59, *n* = 9; TTX/Aβ 96 h recovery = 15.8 ± 1.51, *n* = 8). **b1** Treatment paradigm of PhTx application during HSP. **b2** Graph of normalized mEPSC amplitudes showing that blockade of CP-AMPARs was sufficient to occlude HSP in both the TTX and TTX/Aβ treatment groups (control = 12.5 ± 0.28, *n* = 7; TTX = 17.1 ± 0.52, *n* = 5; TTX + PhTx = 13.1 ± 0.42, *n* = 6; TTX/Aβ = 19.6 ± 0.79, *n* = 6; TTX/Aβ + PhTx = 13.5 ± 0.28, *n* = 6). **c1** Paradigm of PhTX application after the initiation phase of HSP. Neurons were allowed to initiate HSP normally in the presence of TTX and then treated with PhTx ± Aβ. **c2** Graphs show that blockade of CP-AMPARs after typical HSP initiation was sufficient to occlude Aβ-induced over-scaling of HSP (control = 12.4 ± 0.50, *n* = 7; TTX = 17.1 ± 0.65, *n* = 6; TTX → PhTx = 16.9 ± 0.68, *n* = 7; TTX/Aβ = 20.1 ± 0.83, *n* = 6; TTX/Aβ → PhTx = 17.6 ± 0.38, *n* = 8 cells). Mann–Whitney *U* test, * *p* < 0.05, ** *p* < 0.01, *** *p* < 0.001, ns: not significant

Homeostatic regulation serves to restrain synaptic activities within a physiological range from a certain threshold, or a set-point. It is possible that the Aβ-mediated over-scaling of HSP was due to a shift in the homeostatic set-point to a higher level. If so, the synaptic strength may not return to basal levels, even after activity inhibition. To investigate whether TTX/Aβ-mediated HSP was reversible, we removed the treatment medium, washed the neurons and incubated the cells in fresh media after HSP for various lengths of time (Fig. 2a1). 24 h following TTX/Aβ removal, mEPSC amplitudes showed a significant reduction; after 96 h, mEPSC amplitudes of both TTX- and TTX/Aβ-treated neurons returned to basal levels (Fig. 2a2). Therefore, the Aβ-mediated over-scaling of HSP was reversible and seemed to not affect the homeostatic set-point.

To investigate whether Aβ could induce neuronal toxicity during activity deprivation, we performed a cell death assay after 24 hr incubation with Aβ, TTX or TTX/Aβ. Live neurons were treated with propidium iodide (PI) to label dying cells and Hoescht to label all nuclei, with or without a 30uM glutamate challenge to induce excitotoxicity. Quantification of the ratio of PI-positive to Hoescht positive nuclei showed that activity deprivation induced more cell death after a glutamate challenge, however cells treated with Aβ did not showed increased excitotoxicity (Additional file 1: Figure S4).

### CP-AMPARs regulate the Aβ-mediated over-scaling

GluA2-lacking CP-AMPARs are produced and required for the expression of HSP during activity deprivation [25, 30, 39, 53]. To examine the role of CP-AMPARs in Aβ-mediated over-scaling, neurons were treated with TTX or TTX/Aβ with or without the selective CP-AMPAR antagonist, philanthotoxin (PhTx), for 24 h (Fig. 2b1). Inhibition of CP-AMPARs during neuronal inactivity was sufficient to block both TTX- and TTX/Aβ -induced HSP in mEPSCs (B2). Thus, the Aβ-triggered over-scaling shared the same requirement for CP-AMPARs as regular HSP. Previous studies indicate that CP-AMPARs are required for HSP initiation [16, 17, 23, 24] however their role in the expression stage (after initiation) of HSP remains unclear. We therefore treated neurons with TTX for 12 h (for HSP initiation), followed by 12 h incubation with TTX + PhTx (Fig. 2c1). mEPSC recordings from these neurons showed that PhTx application at a later stage did not affect synaptic scaling (Fig. 2c2), indicating that CP-AMPARs were necessary only for the initiation, but not the maintenance of HSP. To investigate whether Aβ-mediated over-scaling was dependent on CP-AMPARs during the later stage of HSP expression, we recorded from neurons that were incubated with TTX alone for 12 h for normal HSP initiation, then with TTX/Aβ + PhTx for the subsequent 12 h (Fig. 2c1). Surprisingly, whereas AMPAR mEPSC amplitudes scaled to levels similar to TTX-mediated HSP, these neurons failed to produce the Aβ-dependent over-scaling of HSP (Fig. 2c2) Additionally, after 24 h treatment with TTX/Aβ, mEPSCs showed a faster decay time (90–10%) indicative of the presence of GluA2-lacking AMPARs (Additional file 1: Figure S5). Because TTX treatment can induce the expression of CP-AMPARs and Aβ may prolong their expression, we examined whether treatment of Aβ alone after 12 h incubation with TTX could produce over-scaling (Fig. 2c1). However, Aβ treatment after a normal initiation of HSP was not sufficient to induce over-scaling similar to 24 hr TTX/Aβ treatment (Fig. 2c2). These findings suggest that CP-AMPARs play a role in the Aβ-mediated over-response of the later expression stage of HSP during periods of inactivity.

### Both GluA2-containing and GluA2-lacking AMPARs contribute to Aβ-induced over-scaling

**Fig. 3:**
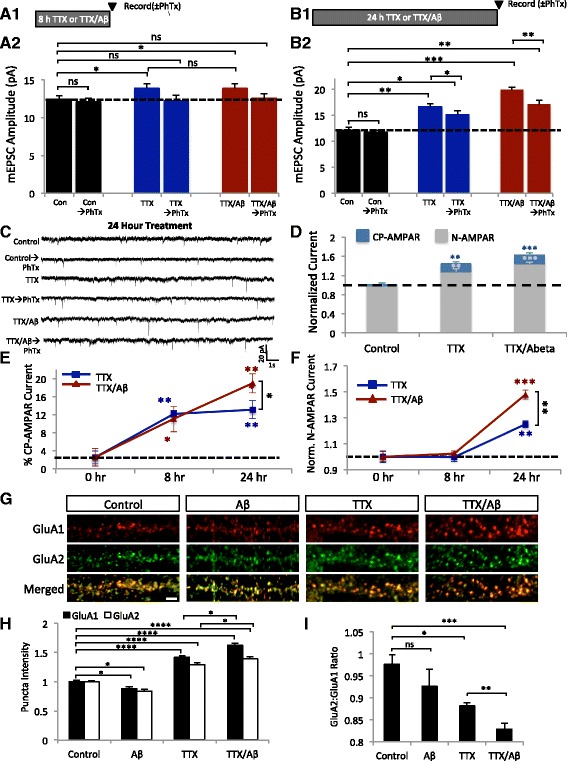
Both CP-AMPAR and N-AMPARs contribute to the Aβ-mediated over-scaling of HSP. **a1** and **b1** Treatment paradigms. mEPSCs were recorded in the presence of PhTx after 8 h (**a1**) and 24 h (**b1**) of TTX or TTX/Aβ incubation. **a2** and **b2** Normalized mEPSC amplitudes after 8h (**a2**) (control = 12.5 ± 0.42, *n* = 10; Con → PhTx = 12.1 ± 0.51, *n* = 10; 8h TTX = 13.9 ± 0.58, *n* = 8; 8h TTX → PhTx = 12.2 ± 0.72, *n* = 8; 8 h TTX/Aβ = 13.9 ± 0.63, *n* = 9; 8 h TTX/Aβ → PhTx = 12.5 ± 0.66; *n* = 9) and 24 h (**b2**) HSP (control = 12.1 ± 0.60, *n* = 9; control → PhTx = 11.7 ± 0.43, *n* =9; TTX = 16.6 ± 0.59, *n* = 8; TTX → PhTx = 15.1 ± 0.76, *n* = 8; TTX/Aβ = 19.9 ± 0.52, *n* = 9; TTX/Aβ → PhTx = 17.1 ± 0.78; *n* = 9). PhTx was only applied to the extracellular recording solution at the time of the recording. **c** Sample traces of mEPSCs after 24 h application of the indicated treatments. **d** Stacked graph of the relative contributions of N-AMPARs (mEPSC amplitude after application of PhTx) *vs.* CP-AMPARs (mEPSC amplitude without PhTx − amplitude with PhTx). Application of Aβ caused an increase in both CP-AMPAR and N-AMPAR components in HSP (control CP-AMPAR = 0.01 ± 0.04, n = 7; control N-AMPAR = 0.98 ± 0.05, *n* = 7; TTX CP-AMPAR = 0.11 ± 0.04, *n* = 7; TTX N-AMPAR = 1.28 ± 0.06, *n* = 7; TTX/Aβ CP-AMPAR = 0.18 ± 0.06, *n* = 6; TTX/Aβ N-AMPAR = 1.44 ± 0.04, *n* = 6). **e** Percentage of the CP-AMPAR component after 8 h and 24 h HSP. Ratio = (amplitude without PhTx- amplitude with PhTx)/(amplitude without PhTx) (TTX 0 h = 2.27 ± 1.42%, *n* = 9; TTX 8 h = 12.14 ± 0.82%, *n* = 9; TTX 24 *h* = 13.09 ± 1.99%, *n* = 8; TTX/Aβ 0 h = 2.52 ± 1.93%, *n* = 8; TTX/Aβ 8 h = 11.05 ± 2.87%, *n* = 10; TTX/Aβ 24 h = 18.99 ± 2.13%; *n* = 10). **f** Changes in the N-AMPAR current after 8 h and 24 h HSP (TTX 0 h = 1 ± 0.04, n = 9; TTX 8 h = 0.99 ± 0.03, *n* = 9; TTX 24 h = 1.26 ± 0.03, *n* = 8; TTX/Aβ 0 h = 1 ± 0.04, *n* = 10; TTX/Aβ 8 h = 1.02 ± 0.03, *n* = 10; TTX/Aβ 24 h = 1.46 ± 0.04; *n* = 10). **g** Immunostainings of surface GluA1 (red) and GluA2 (green) after 24 h incubation with the indicated treatments. Scale bar represents 2 μm. **h** Quantification of changes in surface synaptic intensity of GluA1 and GluA2 (GluA1: control = 1 ± 0.03, *n* = 11; Aβ = 0.88 ± 0.03, *n* =10; TTX = 1.42 ± 0.03, *n* = 11; TTX/Aβ = 1.63 ± 0.04, *n*=11. GluA2: control = 1 ± 0.02, *n* = 10; Aβ = 0.84 ± 0.04, *n* =10; TTX = 1.29 ± 0.03, *n* = 10; TTX/Aβ = 1.39 ± 0.04, *n* =10). Mann–Whitney *U* test, * *p* < 0.05, ** *p* < 0.01, *** *p* < 0.001, **** *p* < 0.0001 ns = not significant

We next sought to characterize the relative contribution of GluA2-containing, normal AMPARs (N-AMPARs) and CP-AMPARs to the mEPSC currents during HSP. We have shown that CP-AMPARs are required for HSP initiation in TTX and TTX/Aβ treated neurons, but whether the additional currents in over-scaling came entirely from CP-AMPARs was not clear. Aβ may facilitate CP-AMPAR expression only transiently during initiation resulting in stronger HSP responses by recruiting more N-AMPARs to synapses during the later stage of HSP. Alternatively, Aβ could simply trigger continuous expression of CP-AMPARs over the entire course, including the early initiation stage and later maintenance stage. To investigate these possibilities, neurons were treated with TTX or TTX/Aβ for 8 or 24 h to allow HSP expression. mEPSC recordings were then conducted either in the presence of PhTx to block CP-AMPARs, or without PhTx to record total AMPAR currents (N-AMPAR + CP-AMPAR). The difference between the two recording conditions would indicate the net contribution of CP-AMPARs. With this approach, we were able to compare the relative contribution of N-AMPARs and CP-AMPARs during the early initiation (8 h) (Fig. 3a1) and the later (24 h) (Fig. 3b1) stages of HSP. After 8 h treatment with TTX or TTX/Aβ, both conditions showed a ~10% increase in mEPSC amplitude over controls, and addition of PhTx during recordings reduced amplitudes back to control levels (Fig. 3a2), indicating that the small increase in mEPSC amplitude during HSP initiation predominantly resulted from addition of CP-AMPARs in both TTX and TTX/Aβ treated neurons. Thus, the Aβ-mediated over-scaling was not due to an earlier, or stronger, onset during the HSP initiation. In contrast, when neurons were treated for 24 h with TTX or TTX/Aβ, addition of PhTx during recordings produced a 23% decrease in mEPSC amplitude in TTX/Aβ treated neurons, compared to a 12% decrease in neurons treated with TTX alone (Fig. 3b2 and 3c). TTX/Aβ treatment therefore resulted in larger amounts of CP-AMPAR expression during the later stage of HSP (Fig. 3d).

Analysis of the ratio of CP-AMPAR to total AMPAR currents showed a larger contribution of CP-AMPARs after Aβ treatment during activity deprivation (Fig. 3e). A comparison of N-AMPAR currents also showed a 20% increase in TTX/Aβ-treated neurons over TTX-treated neurons (Fig. 3f). These results demonstrate that in addition to a modest increase in CP-AMPARs, more N-AMPARs were added during the expression stage of Aβ-mediated HSP. The increase in CP-AMPARs after 24 h inactivity may suggest an Aβ-dependent elongation of the initiation process, which leads to an over-expression of CP-AMPARs and thus an elevated recruitment of N-AMPARs, resulting in HSP over-scaling.

To directly investigate the change in surface AMPAR composition, we immunostained neurons for surface synaptic GluA1 and GluA2 after 24h incubation with Aβ, TTX or TTX/Aβ (Fig. 3g). Aβ incubation alone produced a modest decrease in both surface GluA1 and GluA2 levels (Fig. 3h) with no significant change in the ratio of GluA2:GluA1 (Fig. 3i). With the incubation of TTX, we observed a significant increase in the amount of surface GluA1 and GluA2 compared to control cells (Fig. 3h), as well as a significant decrease in the ratio of GluA2:GluA1 (Fig. 3i) suggesting that both CP-AMPARs and N-AMPARs had been recruited to the synaptic surface. TTX/Aβ treatment led to an increase in both GluA1 and GluA2 levels that were also significantly greater than TTX alone (Fig. 3h). Additionally, we observed a significant decrease in the GluA2:GluA1 ratio after TTX/Aβ treatment compared to TTX treatment alone (Fig. 3i). These findings corroborate our mEPSC recordings and indicate that the Aβ-mediated over-scaling of HSP increases the amount of CP-AMPARs and N-AMPARs at the synaptic surface.

### Aβ produces over-scaling in vivo in mouse visual cortex after visual deprivation

**Fig. 4:**
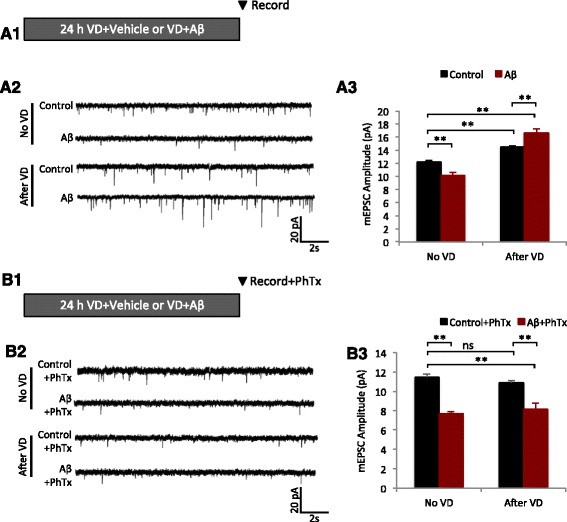
Aβ-mediated over-scaling in vivo requires CP-AMPARs. **a1** Bilateral VD was performed in one month-old mice by sealing the eyelids with black epoxy and the animals were then kept in the dark. Prior to VD, Aβ (10 μg) or vehicle (water) was injected into V1 visual cortex *via* a cannula. Following 24 h VD, brain slices of the visual cortex were prepared for mEPSC recordings to examine synaptic scaling. **a2** Sample mEPSC traces from brain slice recordings after the treatments shown to A1. **a3** Quantification of the data shown in A2 (Control no VD = 12.2 ± 0.23, *n* = 7; Aβ no VD = 10.2 ± 0.48, *n* =6; Control after VD = 14.5 ± 0..19, *n* = 6; Aβ after VD = 16.6 ± 0.63, *n* = 6; 4 mice per condition). **b1** Following 24 h VD paradigm, mEPSCs were recorded from brain slices with, or without PhTx, to determine the contribution of CP-AMPARs to HSP in vivo. **b2** Sample mEPSC traces from brain slice recordings after the treatments shown to B1. **b3** Quantification of the data shown in B2 (Control no VD + PhTx = 11.5 ± 0.33, *n* = 5; Aβ no VD + PhTx = 7.68 ± 0.21, *n* = 5; Control after VD + PhTx = 10.9 ± 0.21, *n* = 6; Aβ after VD + PhTx = 8.2 ± 0.63, *n* = 5; 4 mice per condition). Mann–Whitney *U* test, * *p* < 0.05, ** *p* < 0.01, *** *p* < 0.001, ns = not significant

To determine whether Aβ-mediated over-scaling of HSP could occur in vivo, we adopted a mouse model for HSP in the visual cortex with visual deprivation (VD) [11, 18]. In 1 month-old mice, Aβ (10 μg), or vehicle (water), was injected into V1 visual cortex *via* a cannula, then bilateral VD was conducted and the animals were kept in the dark. Following 24 h VD, brain slices of the visual cortex were prepared for mEPSC recordings to examine synaptic scaling (Fig. 4a1). Consistent with previous studies [18, 31], VD was able to induce a significant homeostatic increase in mEPSC amplitude. We observed a 19% increase in mEPSC amplitude in VD animals compared to controls (Fig. 4a2–a3). In line with our recordings in cultured neurons, Aβ injection alone decreased mEPSC amplitude (Fig. 4a2–a3) and frequency (Additional file 1: Figure S6) compared to vehicle control animals. However, VD + Aβ produced an overshoot in the HSP response. Recordings from VD + Aβ treated animals showed a 36% increase in mEPSC amplitude compared to control, while VD alone without Aβ showed a 19% increase compared to control (Fig. 4a2–a3). Thus, similar to the in vivo findings, Aβ induced an over-scaling in visual cortical neurons in vivo under activity suppression conditions.

### CP-AMPARs are responsible for Aβ-induced over scaling in visual cortex

To investigate the contribution of CP-AMPARs to Aβ-mediated HSP in vivo, we performed visual deprivation for 24 h after injection of Aβ or vehicle into V1 visual cortex and recorded mEPSCs from brain slices in the presence of PhTx (Fig. 4b1). Strikingly, application of PhTx to brain slices from VD + Aβ animals was sufficient to return mEPSC amplitudes to levels similar to control animals without VD (Fig. 4b2–b3). Therefore, in visual cortex in vivo, the elevated mEPSC amplitude during HSP was constituted predominantly of CP-AMPARs. These findings suggest that compared to HSP in cultured neurons, CP-AMPARs are the major component for HSP expression in vivo.

### miR124 is necessary for CP-AMPAR production and Aβ-induced over scaling

**Fig. 5:**
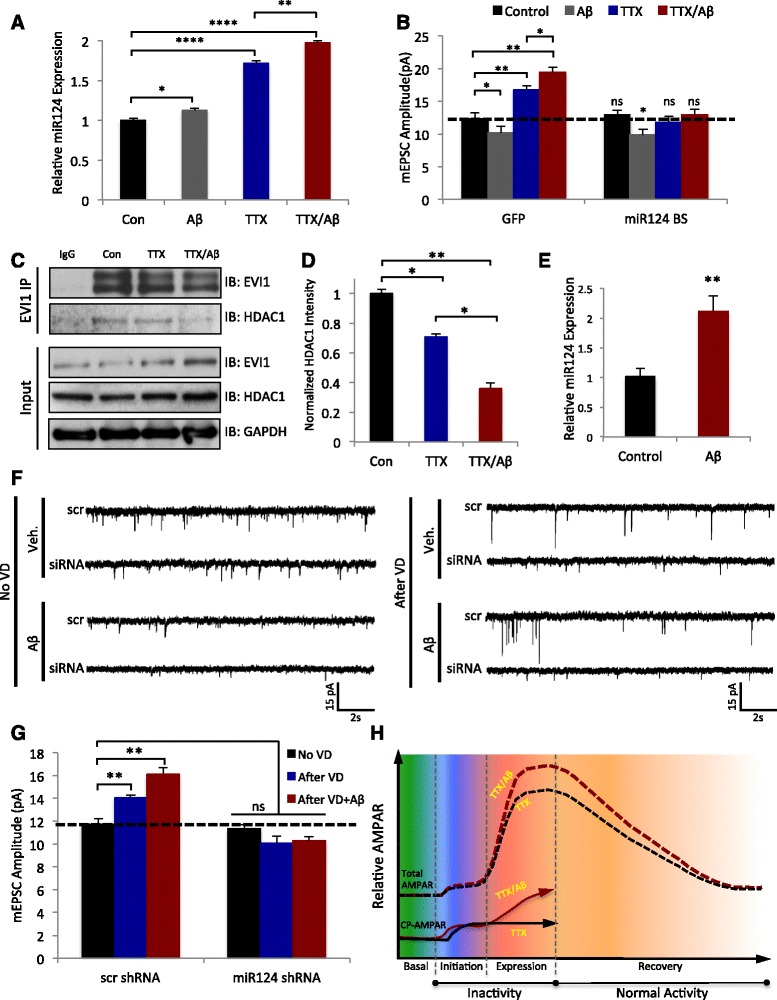
miR124 expression is increased in Aβ-mediated over-scaling due to a decreased EVI1-HDAC1 interaction. **a** MicroRNAs were purified from rat hippocampal lysates treated for 24 h with the conditions shown and miR124 was quantified by qRT-PCR (Control = 1 ± 0.02, *n* = 3; Aβ = 1.12 ± 0.02, *n* =3; TTX = 1.72 ± 0.03, *n* = 3; TTX/Aβ = 1.97 ± 0.02, *n* =3). Values normalized to control, error bars = ± SEM. **b** Hippocampal neurons were transfected with a miR124 BS (containing GFP) or a scrambled BS-GFP for 1 day followed by 24 h treatment with the conditions shown. mEPSC amplitudes were increased in the scrambled BS controls but not in the miR124 BS transfected cells (Control + GFP = 12.33 ± 0.86, *n* = 3; Aβ + GFP = 10.2 ± 0.97, *n* =3; TTX + GFP = 16.8 ± 0.62, *n* = 3; TTX/Aβ + GFP = 19.5 ± 0.74, *n* =3; Control + BS = 12.9 ± 0.74, *n* =8; Aβ + BS = 9.9 ± 0.83, *n* =3; TTX + BS = 11.8 ± 0.86, *n* =9; TTX/Aβ + BS = 12.9 ± 0.86, n =8; 4 mice per condition). **c** Co-immunoprecipitation of EVI1 and HDAC1. Using lysates from rat cortical neurons incubated with the conditions shown for 24 h, EVI1 complexes were isolated by immunoprecipitation and probed for HDAC1. EVI1 co-immunoprecipitated with HDAC1 and the association was further decreased by incubation with TTX/Aβ. **d** Quantification of the co-immunoprecipitation data from C (*n* = 4); HDAC1 was normalized to the immunoprecipitated EVI1 levels. **e** MicroRNAs were purified from mouse cortex 24 h after injection of Aβ (10μg) or vehicle control and miR124 was quantified by qRT-PCR (Control = 1.01 ± 0.13, *n* =10; Aβ = 2.12 ± 0.25, *n* = 10). Values normalized to control, error bars = ± SEM. **f** Sample mEPSC traces from recordings in V1 visual cortex for the treatments shown. **g** Quantification of the traces shown in E. mEPSC amplitudes were increased in the scrambled siRNA controls for VD and VD + Aβ but not in the miR124 siRNA infected cells (no VD + scr siRNA = 11.7 ± 0.52, *n* = 6; VD + scr siRNA = 14.1 ± 0.23, *n* =8; VD + Aβ + scr siRNA = 16.1 ± 0..57, *n* = 5; no VD + miR124 siRNA = 11.4 ± 0.33, *n* =6; VD + miR124 siRNA = 10.1 ± .62, *n* = 5; VD + Aβ + miR124 siRNA = 10.3 ± .36, *n* =6; 4 mice per condition). **h** Model depicting the disruption of HSP by Aβ. During initiation, both TTX and TTX/Aβ show a normal increase in CP-AMPARs (solid lines), however in the presence of Aβ, CP-AMPAR expression continues to increase subsequently driving a further increase in N-AMPARs and total AMPARs (dashed lines) during the expression phase of HSP. Mann–Whitney *U* test, * *p* < 0.05, ** *p* < 0.01, *** *p* < 0.001, **** *p* < 0.001, ns = not significant. Values = mean, error bars = ± SEM

MicroRNA miR124 has been shown to play a key role for the generation of CP-AMPARs [22, 24]. miR124 selectively binds to the 3’-UTR of GluA2 mRNA suppressing protein expression of GluA2. Probably *via* calcium signaling gated by calcium permitting CP-AMPARs, an increase in miR124, and subsequent formation of CP-AMPARs is required for the homeostatic response [24]. To examine miR124 expression, we treated neuronal cultures with TTX, Aβ, or TTX/Aβ for 6h and collected total RNA. qPCR revealed that miR124 expression was increased 12% in neurons treated with Aβ alone, comparing to a 72% increase in neurons incubated with TTX (Fig. 5a). Surprisingly, co-incubation of TTX with Aβ produced a 97% increase in miR124 that was significantly larger than TTX or Aβ alone (Fig. 5a). These results indicate that under a condition of activity suppression, Aβ is capable of dramatically up-regulating miR124 expression in neurons, which presumably leads to the generation of a higher amount of CP-AMPARs for HSP expression.

To examine the necessity of miR124 in Aβ-mediated HSP, we transfected hippocampal neurons with a miR124-binding sponge (BS) construct to neutralize endogenous miR124 [24]. The sponge consisted of two tandem repeats of a sequence complementary to miR124, so that when overexpressed, it would bind and sequester miR124. The miR124-BS has previously been validated to block the initiation of TTX induced HSP [24]. We found that expression of the miR124-BS not only blocked HSP by TTX, but also HSP over-scaling induced by TTX/Aβ, indicating that the up-regulation of miR124 was required for HSP (Fig. 5b), presumably due to a suppression of CP-AMPAR formation. Additionally, expression of the miR124-BS with Aβ showed an ~10% decrease in amplitude, similar to treatment with Aβ + GFP (Fig. 5b).

### Aβ destabilizes the association between EV1 and HDAC1

The transcription factor EVI1 has been shown to regulate miR124 expression by binding to the regulatory region of the miR124 gene to suppress its transcription [12, 49, 57]. Association of EVI1 with the histone deacetylase HDAC1 is crucial for transcriptional repression *via* histone deacetylation [4, 20]. Importantly, a decreased EV1-HDAC1 interaction has been shown to be controlled by neuronal activity and to control miR124 expression [24]. We hypothesized that treatment with Aβ during activity deprivation could further decrease the EVI1-HDAC1 interaction, thereby increasing miR124 levels. We therefore immunoprecipitated EVI1 from lysates of cultured cortical neurons after treatment with TTX or TTX/Aβ for 24 h and probed for HDAC1. Indeed, activity deprivation by TTX caused a reduction in the EVI1–HDAC1 interaction, and association was further reduced with the TTX/Aβ treatment (Fig. 5c and 5d), consistent with the idea that a destabilization of the EVI1-HDAC1 association by Aβ resulted in elevated miR124 expression.

### miR124 is required for Aβ-induced over-scaling in vivo

Next, we wanted to examine whether miR124 was implicated in the aberrant over-scaling in vivo First, we found that following Aβ injection in the visual cortex and 24 h VD, qPCR confirmed a 110% increase in miR124 expression compared to vehicle controls (Fig. 5e). To confirm the role of miR124 in HSP in vivo, we injected miR124 inhibitor virus, or a scrambled control, one month prior to VD experiments to allow for viral expression. Mice were then injected with vehicle or Aβ, into the visual cortex followed by 24 h VD. mEPSC recordings from visual cortical brain slices showed that knockdown of miR124 in vivo blocked VD-induced HSP in both vehicle and Aβ treated animals (Fig. 5f-g), indicating that miR124 expression is necessary for normal and over-scaling of HSP in vivo.

## Discussion

For the first time, we provide evidence indicating a role for Aβ in homeostatic synaptic regulation, a mechanism counter-balancing Hebbian synaptic plasticity and crucial for the stabilization of neuron and neural circuit activity. During normal conditions, activity suppression triggers HSP thereby returning synaptic activity to a physiological level. However, in the presence of Aβ, neuronal inactivity triggered an overshoot in the HSP response, producing an aberrant over-scaling of synaptic strength (Fig. 5h). Our findings strongly support a role of altered homeostatic plasticity in AD, a hypothesis that has previously been proposed by Small [51, 52].

The increased synaptic scaling was caused by a larger and more prolonged expression of CP-AMPARs during the course of HSP. An increase in the generation of CP-AMPARs was observed as a result of an increase in miR124 expression after Aβ treatment, both in rat hippocampal neurons in vivo and mouse visual cortex in vivo. Although miR124 facilitates the expression of HSP, an increase in expression of miR124 alone is not sufficient to induce a homeostatic response [24]. From our findings, Aβ induces a destabilization of the EV1-HDAC1 interaction, thereby allowing for more miR124 transcription and subsequent generation of CP-AMPARs. This could possibly prime the system such that during activity blockade an abnormal overshoot in the HSP response can be triggered. In addition to miR124-dependent generation of CP-AMPARs, Aβ may remove GluA2-containing AMPARs from synapses preferentially thereby promoting the HSP response.

HSP serves to maintain neuronal networks within a physiological range while conserving individual synaptic weights. With an Aβ-mediated over-scaling of HSP, the network could destabilize, or individual synaptic responses could become saturated, thereby disrupting information processing and contributing to memory deficits. Potentially, synaptic over-scaling by Aβ could lead to an increase in the overall excitability in a local neural network. Indeed, epidemiological studies have shown that AD confers an increased risk of seizures and epilepsy, with about a sevenfold increase in seizure incidences in AD patients compared to normal controls [27]. While seizures can appear at any stage of the disease, epileptiform activity is highest in younger AD patients earlier in the disease progression [1, 28]. In AD transgenic mice, elevated levels of Aβ correlate with a higher incidence of seizures before evidence of neuronal loss [45]. Mechanisms that underlie AD-related seizures remain unclear, but HSP over-scaling may represent a causal factor. Additionally, since synaptic release of Aβ is stimulated by neuronal activity, increased synaptic activity could create a positive feedback loop that accelerates AD progression [44, 51, 52].

In line with our findings, HSP of excitatory synapses has been shown to be impaired in neurons lacking presinilin 1 (PS1^−/−^) or neurons expressing a familial AD-linked mutation (PS1^M146V^), which was not dependent on γ-secretase activity and showed impaired phosphatidylinositol 3-kinase (PI3K)/Akt signaling [46]. These findings suggest that other mechanisms may have different effects on HSP in AD and indicate that aberrant synaptic scaling may play a role in familial as well as sporadic AD.

There is a growing body of evidence implicating CP-AMPARs during the onset of synaptic pathology in neurological disorders [21, 35, 47, 60]. Previous analyses of hippocampal postsynaptic density fractions from human AD patients showed an increase in GluA1 levels compared to healthy controls, whereas no changes in NMDAR subunit expression were observed [41], suggesting a selective increase in GluA1-containing receptors in the hippocampus of AD patients. Consistent with this, aberrant CP-AMPAR expression and increased GluA1 phosphorylation have been reported in young mice, prior to any neuropathology, in an AD transgenic mouse model [42]. Together, these findings support a mechanism in which increased CP-AMPAR synaptic insertion during the early stages of AD pathogenesis leads to later synaptic pathologies. In addition, given the unique property that CP-AMPARs have a high permeability to calcium, it is intriguing to postulate an additional role of the CP-AMPARs expressed during HSP in neuronal death during the later stages of AD. Therefore, the CP-AMPAR as well as the HSP regulation cascade could serve as a therapeutic target in the development of new treatments in AD.

## Conclusions

Homeostatic synaptic plasticity (HSP) serves to maintain neuronal and network activity within a physiological range by regulating AMPAR synaptic abundance and thus scaling synaptic activity. We report for the first time that during neuronal inactivity, Aβ application produces an aberrant over-scaling of AMPA receptor (AMPAR)-mediated mEPSCs in both neuronal cultures and in the brain in vivo in a model of visual deprivation. Via stimulating miR124 expression, Aβ results in biogenesis of GluA2-lacking, calcium-permeable AMPARs (CP-AMPARs), which are required for HSP initiation. The aberrant over-scaling effect of Aβ may underlie the unbalanced neuronal network activity and seizures observed in the early phases of AD, as well as neuronal death and cognitive dysfunction in the later phases of AD.

## Additional file

Additional file 1: Figure S1–S6. (PDF 1580 kb)
