# Erratum to: β-Amyloid triggers aberrant over-scaling of homeostatic synaptic plasticity

**DOI:** 10.1186/s40478-017-0423-y

**Published:** 2017-03-09

**Authors:** James Gilbert, Shu Shu, Xin Yang, Youming Lu, Ling-Qiang Zhu, Heng-Ye Man

**Affiliations:** 1 Department of Biology, Boston University, Boston, MA USA; 2 Department of Physiology, School of Basic Medicine, Tongji Medical College, Huazhong University of Science and Technology, Wuhan, 430030 People’s Republic of China; 3 Department of Pathophysiology, School of Basic Medicine, Tongji Medical College, Huazhong University of Science and Technology, Wuhan, 430030 People’s Republic of China; 4 Department of Pharmacology & Experimental Therapeutics, Boston University School of Medicine, Boston, MA USA; 5 The Institute for Brain Research, Collaborative Innovation Center for Brain Science, Huazhong University of Science and Technology, Wuhan, 430030 People’s Republic of China

## Erratum

In the original publication of this article [1], Fig. 4b contains a recording trace which was duplicated. In this Erratum the original Fig. 4b (Fig. 1) and the corrected Fig. 4b (Fig. 2) are published.

**Fig. 1:**
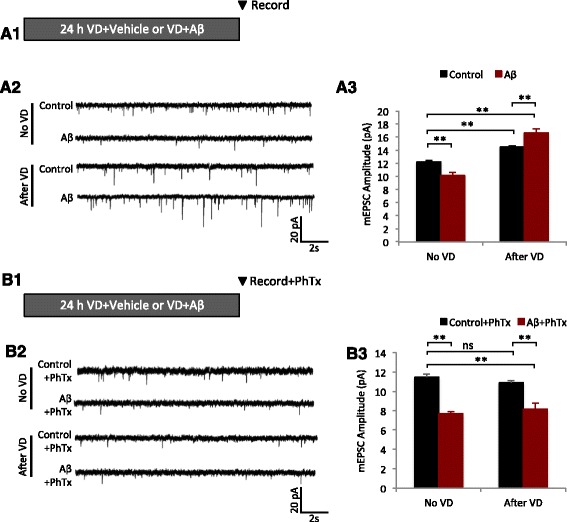
Original version of Fig. 4b as published on 13 December 2016

**Fig. 2:**
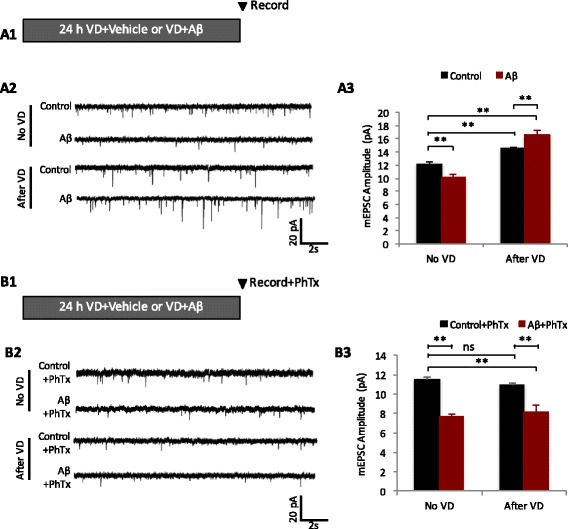
Correct version of Fig. 4b, the second trace has been corrected
