# Supplementary material for: β-Amyloid triggers aberrant over-scaling of homeostatic synaptic plasticity
Source: Acta Neuropathol Commun. 2016 Dec 13;4:131. doi: 10.1186/s40478-016-0398-0 (PMC5154098; doi:10.1186/s40478-016-0398-0)
Supplement: Additional file 1: — Figure S1–S6. (PDF 1580 kb) [file 40478_2016_398_MOESM1_ESM.pdf]

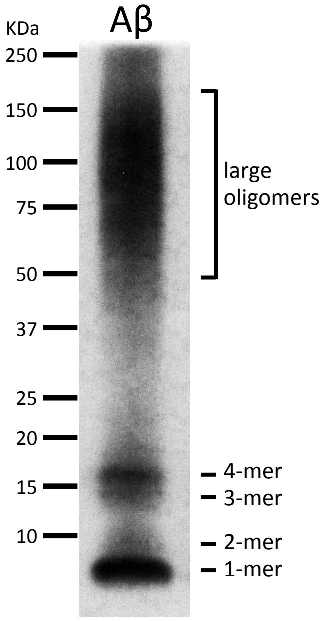

Figure S1. Analysis of Aβ oligomers

Representative western blot of the Aβ 1-42 oligomers used in all experiments. Aβ was prepared from monomer as indicated in Methods.

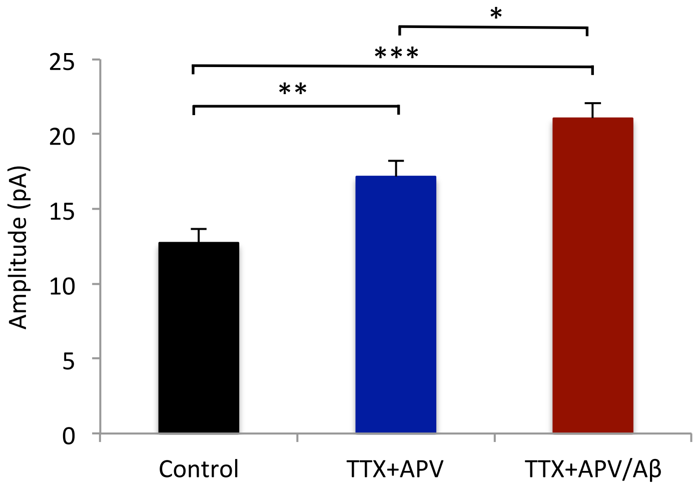

Figure S2. Aβ causes over-scaling in HSP induced by TTX +APV

Quantification of mEPSC amplitude after treatment with TTX +APV ± Aβ (control = 12.7 ± 0.94, n = 9; TTX = 17.2 ± 1.08, n = 7; TTX/Aβ = 21.05 ± 1.05, n = 8). Mann-Whitney U test, * $p < 0.05$, ** $p < 0.01$; *** $p < 0.001$. Values = mean, error bars = ± SEM.

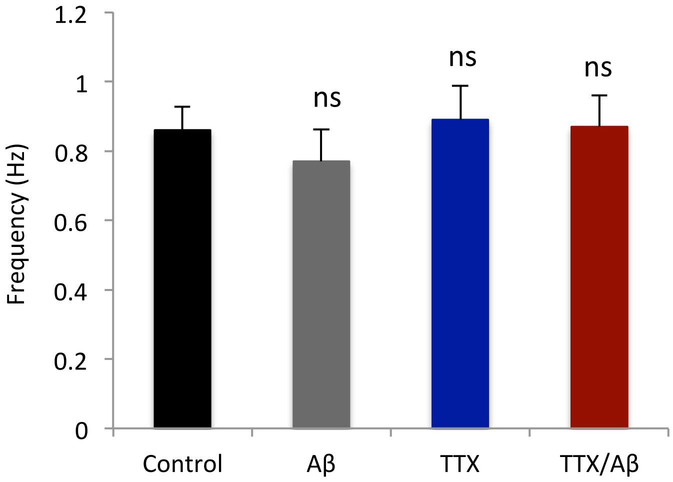

Figure S3. mEPSC Frequency *in vitro*
Quantification of mEPSC frequencies (control = 0.86 ± 0.06, n = 9; Aβ = 0.77 ± 0.08, n = 9; TTX = 0.89 ± 0.09, n = 7; TTX/Aβ = 0.87 ± 0.08, n = 8). Mann-Whitney U test, ns = not significant. Values = mean, error bars = ± SEM.

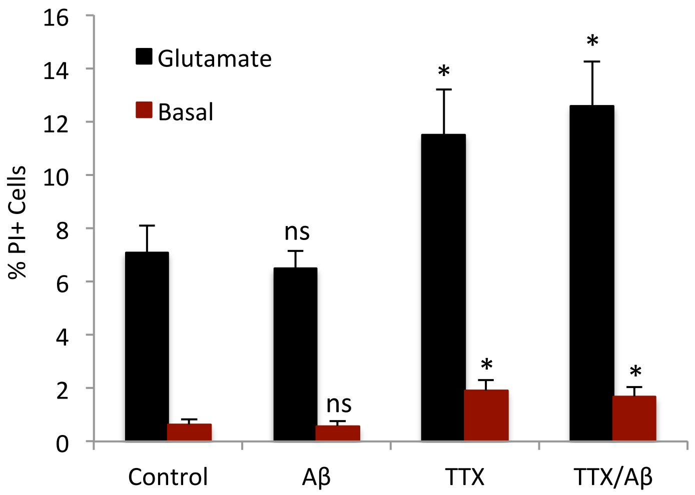

Figure S4. Aβ treatment does not induce toxicity during HSP
Following the indicated HSP paradigms, neurons were treated with propidium iodide (PI) to label dying cells, and Hoescht to label all nuclei. The percentage of PI positive to total Hoescht nuclei was calculated (Basal: control = 0.64% ± 0.2%, n = 10; Aβ = 0.58 ± 0.19, n = 11; TTX = 1.9 ± 0.4, n = 11; TTX/Aβ = 1.69 ± 0.35, n = 12; one-way ANOVA, $p < 0.01$; Glutamate: control = 7.1 ± 1.0, n = 11; Aβ = 6.5 ± 0.66, n = 12; TTX = 11.5 ± 1.71, n = 10; TTX/Aβ = 12.6 ± 1.65, n = 12). Mann-Whitney U test, *$p < 0.05$, ns: not significant. Values = mean, error bars = ± SEM.

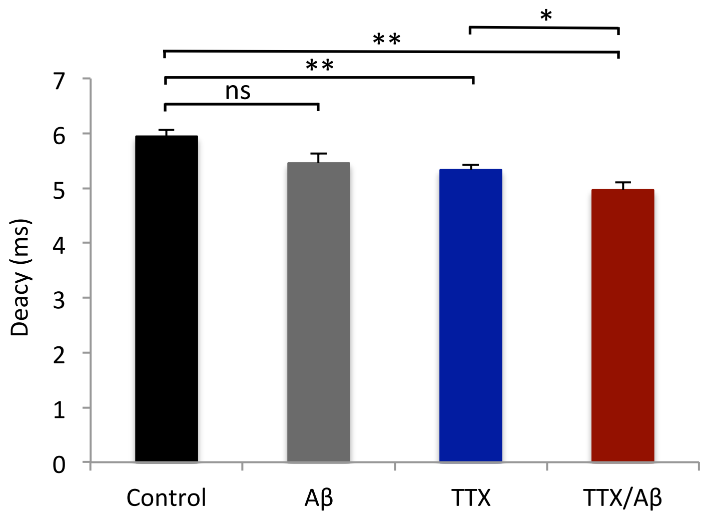

Figure S5. mEPSC decay time

Quantification of mEPSC decay values (control = 5.94 ± 0.12, n = 5; Aβ = 5.55 ± 0.18, n = 5; TTX = 5.33 ± 0.10, n = 5; TTX/Aβ = 4.97 ± 0.14, n = 5). Mann-Whitney U test, $*p < 0.05$, $**p < 0.01$; ns: not significant.

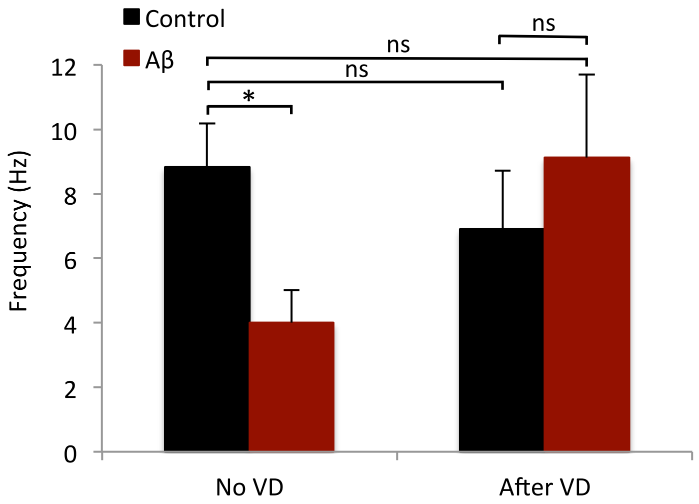

Figure S6. mEPSC frequency *in vivo*

Quantification of mEPSC frequencies (Control no VD = 8.83 ± 1.36, n = 7; Aβ no VD = 4.00 ± 1.01, n = 6; Control After VD = 6.90 ± 1.81, n = 6; Aβ After VD = 9.12 ± 2.58, n = 6). Mann-Whitney U test, *$p < 0.05$, ns = not significant. Values = mean, error bars = ± SEM.
